# Behavioural response of female *Culex pipiens pallens* to common host plant volatiles and synthetic blends

**DOI:** 10.1186/s13071-015-1212-8

**Published:** 2015-11-17

**Authors:** Bao-Ting Yu, Yan-Mei Ding, Jian-Chu Mo

**Affiliations:** Ministry of Agriculture Key Laboratory of Agricultural Entomology, Institute of Insect Sciences, Zhejiang University, 388 Yuhangtang Road, Hangzhou, Zhejiang 310058 China

**Keywords:** *Culex pipiens pallens*, Host plants, Sugar feeding, Volatile compounds, Attractant

## Abstract

**Background:**

Most mosquito species need to obtain sugar from host plants. Little is known about the chemical cues that *Culex pipiens pallens* use during their orientation to nectar host plants. In this study, we investigated the behavioural responses of female *Cx. pipiens pallens* to common floral scent compounds and their blends.

**Methods:**

Behavioural responses of female *Cx. pipiens pallens* to 18 individual compounds at different concentrations were determined in the olfactometer bioassays. A synthetic blend composed of behaviourally active compounds was formulated, and its attractiveness to mosquitoes was tested. Several most attractive compounds constituted a reduced blend, and its attractiveness was tested against the solvent and the full blend, respectively. Mosquito response in the olfactometer was analyzed by comparing the percentages of mosquitoes caught in the two arms by χ^2^ test (observed versus expected).

**Results:**

Fifteen of the 18 compounds were attractive to female *Cx. pipiens pallens* in the dose-dependent bioassays, with the exception of β-pinene, acetophenone and nonanal. (68.00 ± 2.49) % mosquitoes responded to the full blend composed of these 15 compounds on their optimal doses when tested against the solvent, with the preference index at 46.11 ± 3.57. Six individual compounds whose preference indices were over 40 constituted the reduced blend, and it attracted (68.00 ± 1.33) % mosquitoes when tested against the solvent while its preference index was 42.00 ± 3.54. When tested against the full blend simultaneously in the olfactometer, the reduced blend could attract (45.00 ± 2.69) % of released mosquitoes, which was as attractive as the full blend.

**Conclusions:**

Our results demonstrate that female *Cx. pipiens pallens* is differentially attracted by a variety of compounds at different concentrations. Alteration of the concentration strongly affects the attractiveness of the synthetic blend. Several floral scent volatiles might be the universal olfactory cues for various mosquito species to locate their nectar host plants, which could be potentially used in trapping devices for surveillance and control of them.

**Electronic supplementary material:**

The online version of this article (doi:10.1186/s13071-015-1212-8) contains supplementary material, which is available to authorized users.

## Background

*Culex pipiens pallens* Coquillett, the most prevalent *Culex* species in Northeastern Asia, is the primary vector of lymphatic filariasis and epidemic encephalitis [1, 2]. The application of large quantities of insecticides in controlling this mosquito species has resulted in the resistance of *Cx. pipiens pallens* to most types of insecticides, which makes their control increasingly difficult [3]. Therefore, the development of odour-bait technology has been advocated as novel and more environmentally friendly methods against mosquitoes, and it will be an important component of integrated vector management strategies [4, 5].

Sugar feeding is a common behaviour of adult mosquitoes, and most mosquito species need to obtain sugar as a source of energy [6, 7]. Floral and extrafloral nectaries are the primary sugar sources for mosquitoes [6]. Mounting evidences indicate that feeding on different kinds of nectar sources affects the flight performance, survival and fecundity of adult mosquitoes [8–10]. Plant-sugar feeding behaviours of *Anopheles gambiae*, *Aedes albopictus* and *Cx. pipiens* have been observed in the laboratory and field experiments [11–13], and the results reveal that mosquitoes show differential preference for certain host plants [11–16]. However, the cues responsible for the differential attraction of mosquitoes to them are not well understood [6, 17].

Previous studies indicated that visual cues and volatile compounds released by flowers were important cues for mosquitoes to discriminate and locate nectar host plants [6, 7]. Various volatile compounds have been identified from the preferential plant hosts of some mosquito species, and most of them are aromatics, monoterpenes and fatty acid derivatives [16–22]. Several individual compounds could elicit electrophysiological and behavioural responses in *An. gambiae*, *Cx. pipiens molestus* and *Ae. aegypti*, and the attractiveness of compound blends on basis of their mean ratios in the scent profiles to mosquitoes has been evaluated [16, 17, 19–21]. Moreover, the combination of vertebrate kairomones and synthetic plant odours as attractants in traps has shown a synergistic attraction in trapping outdoor populations of malaria vectors [4], which exhibits extensive application prospect in surveillance and control of mosquito populations.

Floral volatile organic compounds are mainly composed of four chemical groups: aromatics, monoterpenes, sesquiterpenes and fatty acid derivatives [23]. In the present study, we investigated the behavioural responses of *Cx. pipiens pallens* females to 18 individual compounds from different groups which are distributed in the volatiles of mosquitoes’ host plants in the dual-choice olfactometer. The attractiveness of a full blend composed of 15 behaviourally active compounds was also tested. Six most attractive compounds were mixed and its attractiveness was tested against the solvent and the full blend, respectively. Our study demonstrated that several odours might be universal chemical cues mediating mosquitoes’ orientation to host plants. By optimizing the dose and constituent of their mixture, we aimed to develop a synthetic floral odorant blend which could be used as attractants in traps.

## Methods

### Mosquitoes

Mosquitoes used in this study were collected from water pools in the urban residential areas in Hangzhou, Zhejiang, China, and had been kept in insectary over 50 generations. They were reared at 25 ± 1 °C with a photoperiod of 14:10 (L:D) and 75 % relative humidity. The adults were maintained on a diet of mouse blood for 3 consecutive days since the 5th day after emergence, with 5 % sucrose solution continuously available. Fully engorged females were allowed to lay eggs on oviposition cups (5 cm diameter, 7 cm depth) inside the cages. Eggs were transferred into plastic basins (30 cm diameter, 11 cm depth) filled to a depth of 8 cm with dechlorinated tap water, and commercial rat food was provided for mosquito larvae. Pupae were collected and transferred to mesh-covered cages (35 × 35 × 35 cm). Newly emerged female adults intended for use in bioassays were given access to water only. Experiments were conducted 12–24 h after emergence.

### Ethics statement

Although mosquitoes were fed upon the mouse blood, this was performed only as a routine method for mosquito maintenance and it was approved by Zhejiang University ethical review committee.

### Chemicals

According to the previous reports on the electrophysiological and behavioural responses of different mosquito species to their host plant volatiles [16–21], we selected 18 floral scent compounds as odorants in the behavioural assays. (*E*)-β-ocimene (≥90 %), α-pinene (98 %), β-pinene (98 %), D-limonene (95 %), linalool (98 %),benzaldehyde (>98 %), phenyl acetaldehyde (95 %), benzyl alcohol (>99 %), methyl salicylate (≥99.5 %), hexanol (98 %), (*Z*)-3-hexen-1-ol (98 %), (*Z*)-3-hexenyl acetate (98 %), hexanal (97 %), (*E*)-2-hexenal (98 %), nonanal (96 %) and pentane (>99 %) were purchased from Shanghai Aladdin Reagent Co., Ltd., China. Phenylethyl alcohol (>98 %) and acetophenone (analytical reagent) were purchased from Sinopharm Chemical Reagent Co., Ltd., China. Linalool oxide (≥97 %, mixture of isomers) was purchased from Sigma-Aldrich.

### Dual-choice olfactometer assays

**Fig. 1:**
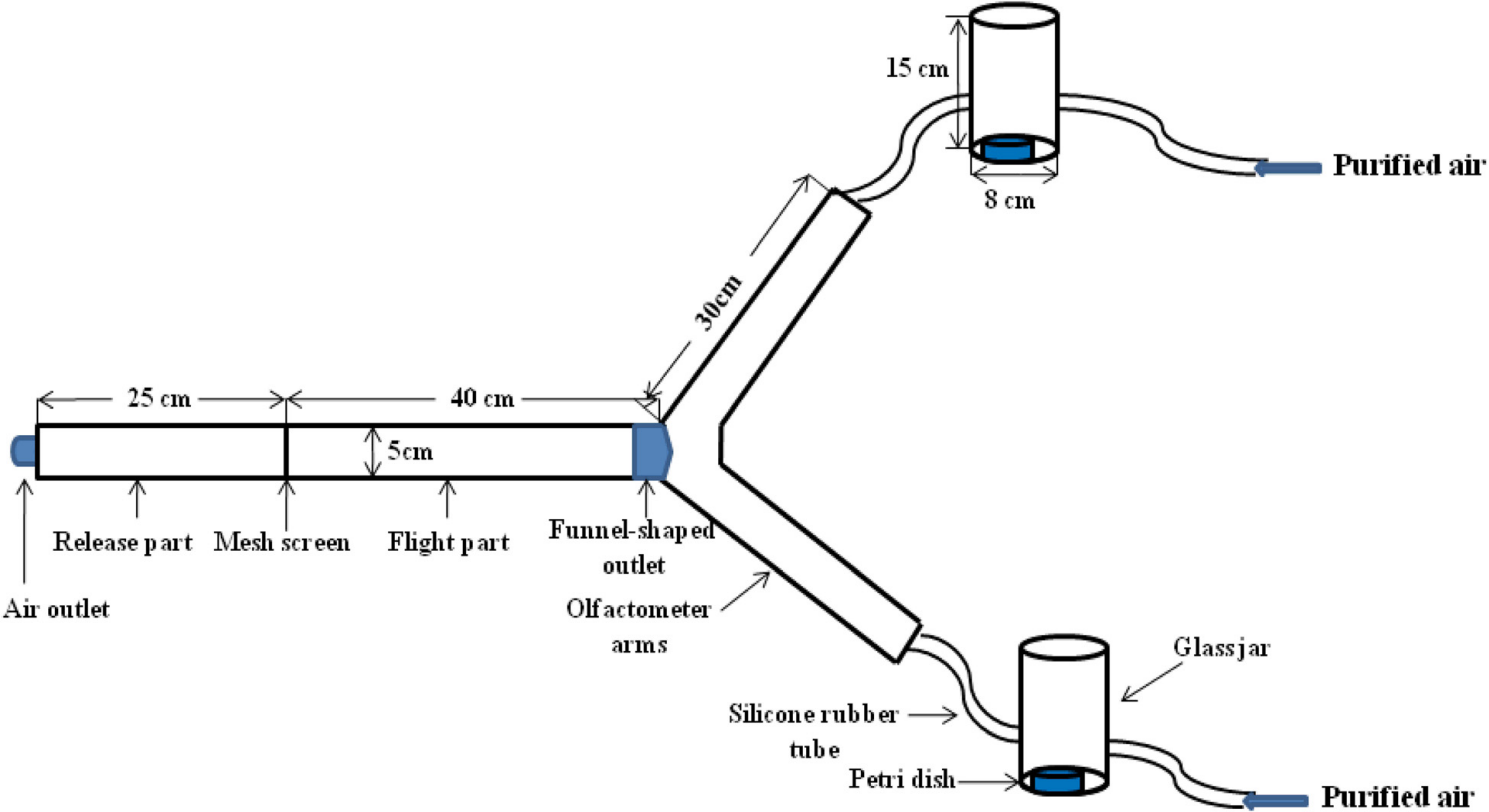
A schematic drawing of the Y-tube olfactometer (not drawn to scale). Dental rolls containing the stimulus and the solvent were placed in the petri dishes, respectively. Mosquitoes entered the flight part when the fabric mesh screen was removed, and they would make the behavioural choice at the funnel-shaped outlet

All behavioural assays of female *Cx. pipiens pallens* to the individual compounds and their synthetic blends were conducted in a modified dual-choice Y-shape olfactometer, as shown in Fig. 1. The olfactometer was made of glass and it consisted of three parts: the release part, the flight part and the olfactometer arms. The release part (diameter 5 cm, length 25 cm) with a demountable glass air outlet was at the downwind end of the olfactometer. To allow the mosquitoes to acclimatize in the release part prior to each trial, a demountable fabric mesh screen was placed between the release part and the flight part. A built-in polycarbonate funnel-shaped outlet (length 4 cm, narrower opening upwind with diameter 2 cm) was located at the upwind of the flight part (diameter 5 cm, length 40 cm) to avoid mosquitoes passing through the tunnel hastily. The angle between two olfactometer arms (diameter 5 cm, length 30 cm) was 90°. Odorants from the glass jars (diameter 8 cm, height 15 cm) entered the olfactometer arms through two rubber plugs (not shown in the schematic drawing), which were inserted on the upwind of the arms, respectively.

Eight doses of each compound were prepared at a concentration of 0.25, 0.5, 0.75, 1, 1.25, 1.5, 1.75 and 2 ug/mL in pentane. The stimulus and the solvent were released by placing 550 ul onto 100 mg medical dental rolls in the petri dishes, respectively. They were left for 10 min at room temperature to allow the solvent to evaporate, and they would be placed into the glass jars, respectively.

Bioassays were conducted during the first 2 h of the dark period, and one 15-W red fluorescent bulb was placed above the olfactometer to illuminate the test arena evenly [17]. The temperature and humidity were adjusted to 25–28 °C and 65–75 %, respectively. Air flow from a compressed air pump was firstly charcoal-filtered and humidified by passing through the distilled water. The purified air entered each glass jar at a flow rate of 0.7 m^3^/h, and the wind speed at the centre of the flight part was 10 cm/s. Groups of 10 robust females were released into the introduce part for 10 min to acclimatize prior to each test. Dental rolls containing the stimulus and the solvent were then placed into the glass jars simultaneously. Mosquitoes in the introduce part were released 30 s later by removing the mesh screen, and the numbers of mosquitoes in two olfactometer arms were counted 5 min later, respectively. Mosquitoes in the olfactometer were removed immediately by an aspirator after each trial. The trials for each dose of individual compounds were replicated 10 times. Mosquitoes and dental rolls were replaced with new ones in each trial. The positions of the treatment and control arms were alternated between trials to eliminate positional biases. All equipments were washed by ethanol and placed in an oven at 100 °C for 6 h. Surgical gloves were worn during the experiments to avoid contamination.

According to the attractiveness of the 18 individual compounds at different concentrations tested above, a full-component blend comprised of optimal doses of 15 individual behaviourally active compounds was prepared at different concentrations (4-fold, 2-fold, 1, 0.5 dilution, 0.25 dilution). The attractiveness of the full blend at different concentrations was tested against the solvent in the olfactometer, respectively. The bioassay procedures were the same as described above.

In order to simplify the constituent of the full-component blend, a reduced blend composed of the 6 most attractive compounds at their respective optimal concentrations was prepared. Its attractiveness to female *Cx. pipiens pallens* was tested against the solvent and the full blend in the dual-choice assays, respectively. All the bioassays were carried out as described above and replicated 10 times.

### Statistical analysis

The percentages of mosquitoes responding to the test odours at each concentration and its control odour were calculated respectively when the trials were replicated 10 times. For each compound/blend at each concentration, the χ^2^ observed versus expected test (based on the percentages of responding mosquitoes in the olfactometer arms) was used to assess the attractiveness of the tested odours with the significance threshold at 0.05 [19]. A preference index (hereafter referred to as PI) was also used to evaluate the attractiveness of the individual compounds and their blends tested in the dual-choice bioassays. It was calculated according to the formula:

$$ \mathrm{PI} = \left[\left(\mathrm{N}\mathrm{P}\hbox{-} \mathrm{N}\mathrm{C}\right)/\left(\mathrm{N}\mathrm{P}+\mathrm{N}\mathrm{C}\right)\right]\times 100 $$

where NP is the number of mosquitoes responding to the test odours and NC is the number of mosquitoes responding to the control odours [17, 19]. A positive value indicates a majority of the mosquitoes responding to the tested odorants, while a negative value indicates the converse [17]. All statistical analysis was carried out using SPSS v. 16 (SPSS Inc, Chicago, USA).

## Results

### Behavioural responses of *Cx. pipiens pallens* to individual compounds

When the selected 18 compounds were prepared at concentrations ranging from 0.25 ug/mL to 2 ug/mL, 15 compounds were attractive to female *Cx. pipiens pallens* compared to the solvent, with the exception of β-pinene, acetophenone and nonanal (Table 1, see Additional file 1: Figure S1 for release rates). Of the tested monoterpenes, α-pinene and D-limonene were only attractive to mosquitoes at the concentrations of 0.5 ug/mL and 0.25 ug/mL, respectively (Table 1, Additional file 2: Figure S2A). Linalool and linalool oxide were optimally attractive at 0.75 ug/mL and 0.5 ug/mL, respectively (Table 1, Additional file 2: Figure S2A). (*E*)-β-ocimene was attractive to mosquitoes in a dose-dependent manner, and its optimal dose was 1 ug/mL (Table 1, Additional file 2: Figure S2A).

**Table 1:** Percentage of mosquitoes responding to 18 individual compounds on their optimal doses

Compound (dose, ug/mL)	Percentage of mosquitoes responding to compound (Mean ± SE)	Percentage of mosquitoes responding to solvent (Mean ± SE)	Preference index (Mean ± SE)	*P*-value
(*E*)-β-Ocimene (1)	74.00 ± 3.40	23.00 ± 3.30	52.73 ± 7.10	<0.01
α-Pinene (0.5)	57.00 ± 2.60	33.00 ± 3.50	23.66 ± 6.63	<0.05
β-Pinene (0.75)	55.00 ± 3.73	38.00 ± 3.89	18.44 ± 7.80	>0.05
D-Limonene (0.25)	60.00 ± 3.33	35.00 ± 4.01	26.89 ± 7.67	<0.05
Linalool (0.75)	68.00 ± 2.91	29.00 ± 2.77	40.22 ± 5.51	<0.05
Linalool oxide (0.5)	60.33 ± 3.33	37.00 ± 3.00	23.56 ± 6.15	<0.05
Benzaldehyde (1.5)	69.00 ± 2.77	27.00 ± 2.13	43.61 ± 4.45	<0.01
Phenyl acetaldehyde (0.5)	64.00 ± 2.67	35.00 ± 2.69	38.56 ± 4.29	<0.01
Benzyl alcohol (1)	62.00 ± 4.90	33.00 ± 4.58	37.11 ± 9.40	<0.05
Phenylethyl alcohol (0.75)	71.00 ± 4.33	26.00 ± 3.71	46.00 ± 7.86	<0.01
Methyl salicylate (1.25)	61.00 ± 2.77	35.00 ± 3.42	38.00 ± 8.62	<0.05
Acetophenone (0.25)	56.00 ± 2.67	41.00 ± 2.77	15.56 ± 5.50	>0.05
Hexanol (1)	65.00 ± 2.24	29.00 ± 2.33	40.00 ± 7.42	<0.01
(*Z*)-3-Hexen-1-ol (0.5)	62.00 ± 3.27	26.00 ± 2.67	40.72 ± 6.02	<0.01
(*Z*)-3-Hexenyl acetate (1)	59.00 ± 2.77	37.00 ± 2.60	22.89 ± 5.20	<0.05
Hexanal (0.75)	61.00 ± 2.33	37.00 ± 2.13	24.44 ± 4.29	<0.05
(*E*)-2-Hexenal (1)	65.00 ± 3.42	29.00 ± 3.48	38.44 ± 7.01	<0.01
Nonanal (0.5)	54.00 ± 2.21	40.00 ± 2.98	15.39 ± 5.08	>0.05

The trials for each dose of each compound were replicated 10 times, and the percentage of mosquitoes responding to the compounds and their pairwise control were calculated and compared by χ^2^ test (observed vs. expected)

All the selected aromatic compounds were attractive to *Cx. pipiens pallens* females, with the exception of acetophenone. Phenyl acetaldehyde was attractive to mosquitoes at 0.5 ug/mL (Table 1, Additional file 2: Figure S2B). The optimal dose of methyl salicylate for attraction was 1.25 ug/mL (Table 1, Additional file 2: Figure S2B). Multiple concentrations of benzaldehyde, benzyl alcohol and phenylethyl alcohol attracted significantly more *Cx. pipiens pallens* than the solvent, and they were optimally attractive at 0.5 ug/mL, 1ug/mL and 0.75 ug/mL (Table 1, Additional file 2: Figure S2B), respectively.

Among the 6 tested fatty acid derivatives, female *Cx. pipiens pallens* were attracted by 5 different compounds, with the exception of nonanal (Table 1, Additional file 2: Figure S2C). (*Z*)-3-hexenyl acetate and (*E*)-2-hexenal were both attractive to mosquitoes at 0.75 ug/mL and 1 ug/mL, with the optimal dose at 1 ug/mL (Table 1, Additional file 2: Figure S2C). (*Z*)-3-hexen-1-ol and hexanal were attractive to mosquitoes at low concentrations, and they were optimally attractive at 0.5 ug/mL and 0.75 ug/mL (Table 1, Additional file 2: Figure S2C), respectively. The attractiveness of hexanol to mosquitoes varied between different concentrations, with the optimal dose at 1 ug/mL (Table 1, Additional file 2: Figure S2C).

### Attraction of *Cx. pipiens pallens* to the behaviourally active compound blends

**Fig. 2:**
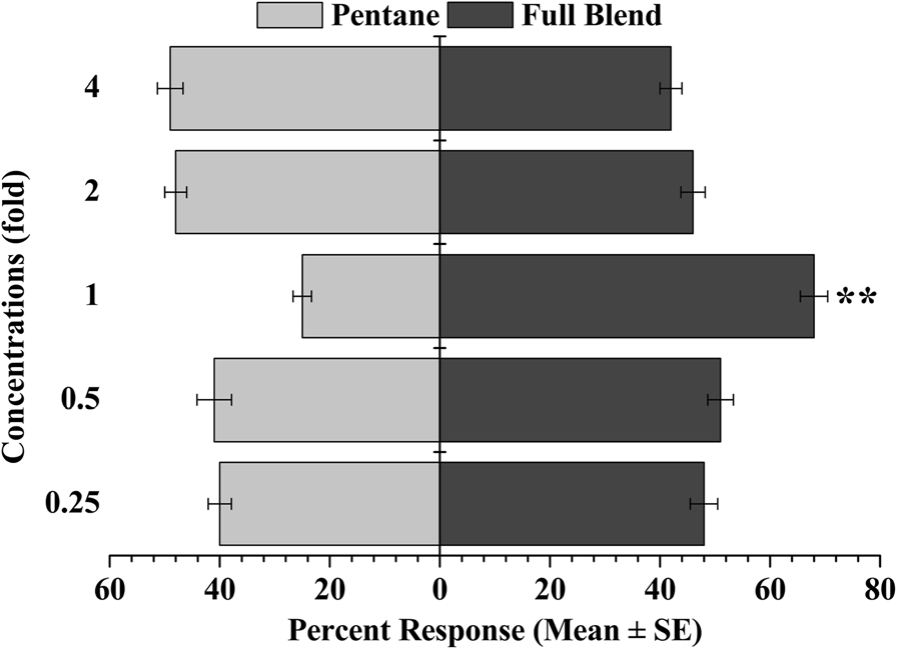
Percent of *Cx. pipiens pallens* responding to a mixture of 15 compounds with different dilutions. Asterisks (**) denote significant differences at *P* < 0.01 by χ^2^ test (observed vs. expected)

The mixture composed of these 15 behaviourally active compounds at different concentrations was formulated, and the results showed that mosquitoes responded to the compound blend in a dose-dependent manner. (68.00 ± 2.49) % *Cx. pipiens pallens* females were strongly attracted by the blend composed of compounds on their optimal doses (χ^2^ = 8.05, df = 1, *P* < 0.01), with the PI at 46.11 ± 3.57 (Fig. 2, see Additional file 3: Figure S3 for release rate). When their concentrations decreased to 50 % and 25 % of the optimal doses respectively, the attractiveness of the blend to mosquitoes decreased and was not significantly different from that of the solvent (χ^2^ = 1.66, df = 1, *P* > 0.05 and χ^2^ = 1.83, df = 1, *P* > 0.05, respectively) (Fig. 2). When the concentrations increased to twice and 4-fold of the optimal doses respectively, most of the tested mosquitoes responded to the solvent rather than the compound blend (χ^2^ = 0.184, df = 1, *P* > 0.05 and χ^2^ = 1.25, df = 1, *P* > 0.05, respectively) (Fig. 2).

### Attraction of *Cx. pipiens pallens* to the reduced blend

**Fig. 3:**
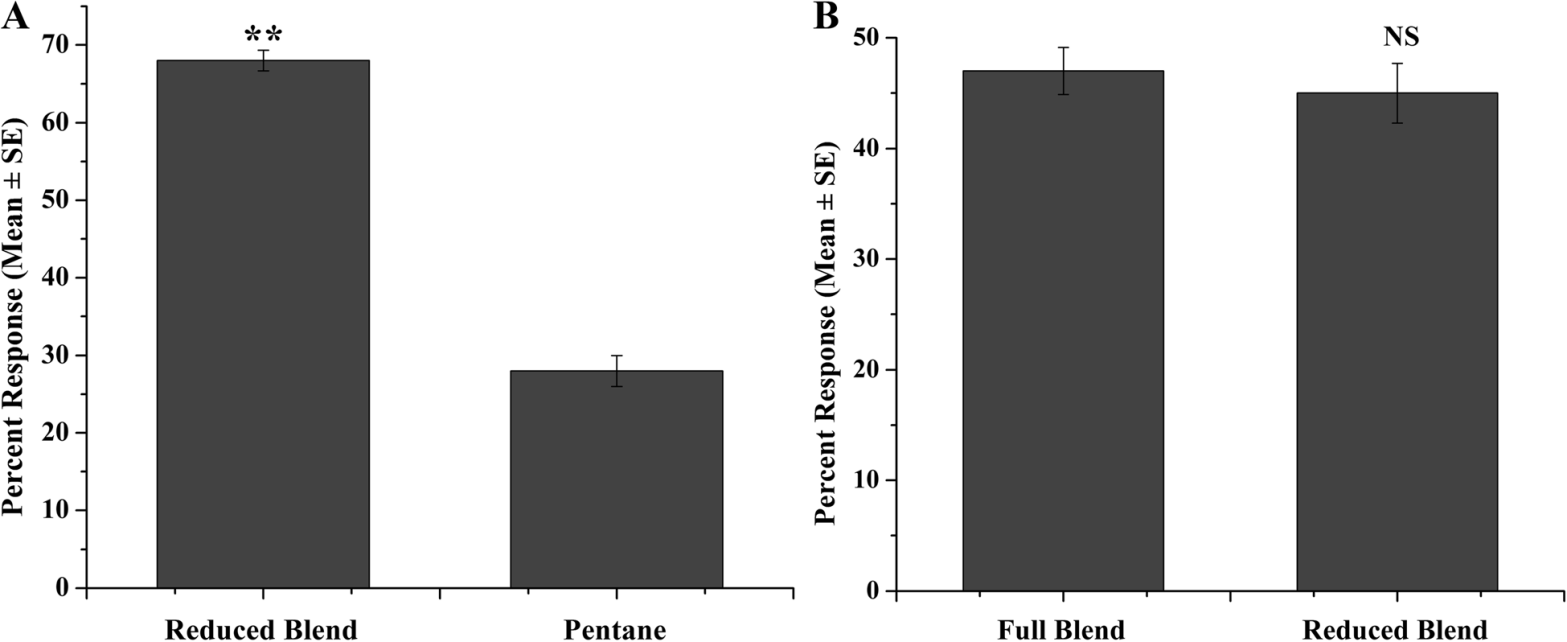
Percent of mosquitoes responding to the reduced blend against the solvent (**a**) and the full blend (**b**). Asterisks (**) denote significant differences at *P* < 0.01 by χ^2^ test (observed vs. expected). NS denotes no significant difference

In attempt to simplify the constituent of the full blend without lowering attraction, we prepared a reduced blend composed of 6 compounds whose mean preference indices were over 40 on their optimal doses (see Additional file 3: Figure S3 for release rate), namely, 1 ug/mL (*E*)-β-ocimene, 1.5 ug/mL benzaldehyde, 1ug/mL hexanol, 0.75 ug/mL phenylethyl alcohol, 0.5ug/mL (*Z*)-3-hexen-1-ol and 0.75 ug/mL linalool. When the reduced blend was tested against the solvent in the olfactometer, (68.00 ± 1.33) % mosquitoes responded to the reduced blend while (28.00 ± 2.00) % mosquitoes responded to the solvent, and mosquitoes were significantly attracted by the reduced blend than the solvent (PI = 42.00 ± 3.54;χ^2^ = 8.64, df = 1, *P* < 0.01) (Fig. 3a). When tested against the full-component blend, the reduced blend was as attractive as the full-component blend (χ^2^ = 0.005, df = 1, *P* > 0.05), with (45.00 ± 2.69) % of released mosquitoes responding to the reduced blend, compared to (47.00 ± 2.13) % for the full-component blend (Fig. 3b).

## Discussion

In the present study, we modified the Y-tube olfactometer and tested the behavioural response of female *Cx. pipiens pallens* to common host plant volatiles and their synthetic blends. The results showed that most mosquitoes could respond to the tested odours in the olfactometer in 5 min, indicating it was an excellent device for testing the behavioural responses of mosquitoes to volatile compounds. Besides, consistent with the previously reported results in other mosquito species [17–21], our results showed that *Cx. pipiens pallens* females could discriminate and respond to different floral scent odours, and their behavioural responses were closely related to the concentrations of the tested compounds. Most individual compounds were attractive to *Cx. pipiens pallens* at the lower end of concentration range, and there was a clear repellent effect for many of the compounds at the higher doses. Increased concentrations or dilutions of the full blend significantly affected the attractiveness of the blend, which indicated the concentration dependent reversal of response.

Previous reports have demonstrated that mosquitoes show differential preference to various host plants in the laboratory and field experiments [14–16], whereas how mosquitoes successfully select and locate their nectar host plants is still not well understood. The structure of inflorescence, the accessibility of sugar and its related nutritional benefits from plants are considered to be important factors influencing mosquitoes to select different flowering plants and seed pods as their sugar sources [6, 10, 16, 24], whereas the color of flowers as well as the floral scent compounds is thought to be important cues for mosquitoes to discriminate and locate various plants [6]. The compounds tested in the present study have been identified from volatiles and pentane extracts of *Silene otites* (Caryophyllaceae), *Asclepias syriaca* (Asclepiadaceae) and other host plants of mosquitoes, and most of them are electrophysiologically and/or behaviourally active to various mosquito species [17–21]. In the present study, newly emerged female *Cx. pipiens pallens* were differentially attracted by these compounds, which indicated that several volatile compounds might be universal olfactory cues in various mosquitoes’ orientation to sugar sources [7, 22].

Most of the selected compounds were behaviourally active to female *Cx. pipiens pallens* when their concentrations ranging from 0.25 ug/mL to 2 ug/mL, with the exception of β-pinene, acetophenone and nonanal. β-pinene is a common constituent of floral scents, and it has been detected in the volatiles of the flowering plants which are attractive to *An. gambiae* and *Cx. pipiens* [16, 17, 21]. β-pinene elicits strong electrophysiological response in *An. gambiae*, but consistent with the present results, it is not attractive to *An. gambiae* either [17]. Acetophenone is distributed in the floral scent profiles of *S. otites*, and it elicits weak electrophysiological response in *Cx. pipiens moles*tus [19, 20]. In the present study, *Cx. pipiens pallens* were not attracted by acetophenone when it was prepared at the concentration of 0.25–0.75 ug/mL, and it exhibited repellent effect when prepared at 1.00–1.75ug/mL. However, the result was contrary to that demonstrated in *Cx. pipiens molestus*, which is highly attractive to acetophenone [19]. Nonanal has been detected in the volatiles of *Senna occidentalis* flowers and pentane extracts of *A. syriaca* flowers, both of whom are attractive to *An. gambiae* [16, 21]. However, the synthetic blend of its host plant volatiles is as attractive as the reduced blend without nonanal in the subtractive bioassays, which indicates that nonanal does not contribute to the attractiveness of the blend of plant volatiles [21]. Besides, nonanal is one important olfactory cue for mosquitoes searching for blood hosts [25, 26], and the olfactory receptors of nonanal have been identified in *Cx. pipiens quiquefasciatus* [26]. In the present study, newly emerged *Cx. pipiens pallens* were not attracted by nonanal at low concentrations and were repelled by it at high concentrations. Thus, we speculate that nonanal may have important roles in mediating female mosquitoes’ searching for blood hosts rather than sugar meals.

In the present study, we formulated a reduced blend composed of six compounds with highest average preference index, and its attractiveness to mosquitoes was tested against the solvent and the full blend, respectively. The results showed that the full blend and the reduced blend could attract (68.00 ± 2.49) % and (68.00 ± 2.13) % mosquitoes when tested against the solvent respectively. When tested against each other, the full blend was as attractive as the reduced blend (percent response: 47 % versus 45 %). However, the mean preference indices of the full blend and the reduced blend were 46.00 and 42.00 respectively, which were higher than that of most individual compounds but similar to or lower than that of (*E*)-β-ocimene and phenylethyl alcohol (mean PI: 52.73 and 46.00, respectively). The similar results have also been observed in the bioassays of *Cx. pipiens molestus* to synthetic blends, where a mixture of the four most attractive compounds attracts more mosquitoes than the mixture of 14 compounds but is as attractive as phenyl acetaldehyde [19]. Therefore, in the present study, existence of other compounds in the blends could not significantly strengthen the attractiveness of the blends, and the lack of additive effect suggests that the reduced blend could be further simplified without loss of attraction.

Based on the sugar feeding behaviour of adult mosquitoes, the newly developed attractive toxic sugar baits (ATSB) have been successfully applied in controlling a variety of mosquito species [27–29]. As part of the ATSB optimization process, an increasing number of attractive flowering plants and seed pods have been identified as potential sugar sources for a variety of mosquito species [13–16], but little is known about the olfactory basis of floral preference of mosquitoes among their nectar host plants. The present study exploited the attractiveness of synthetic floral volatile compounds to *Cx. pipiens pallens*, which could be potentially used in trapping devices for surveillance and control of mosquito populations. Compared with the vertebrate kairomone-based attractants, phytochemical attractants could attract both males and females in all gonotrophic states [7]. Given that different mosquito species might use different chemical cues for orientation to nectar host plants, and they usually visit multiple flowering plant species for sugar feeding, more volatile compounds that are behaviourally active to mosquitoes remain to be exploited. Besides, how to optimize the release rate, to combine the kairomones with phytochemicals and to develop the trap devices deserve further research to maximize the capture rate [7].

## Conclusions

This study demonstrates that newly emerged female *Cx. pipiens pallens* respond differentially to volatile floral scent compounds and their blends at different concentrations. Alteration of the constituents and concentrations significantly affects the attractiveness of the synthetic attractant. Additional studies are required to develop its attractiveness in trap devices for surveillance and control of mosquitoes.

## Additional files

Additional file 1: Figure S1. Olfactometer release rates for optimal doses of 18 synthetic individual compounds. (JPG 2888 kb) Additional file 2: Figure S2. Percent of mosquitoes responding to 18 individual compounds at different concentrations from monoterpenoids (A), benzenoids (B) and fatty acid derivatives (C). Asterisks (*) denote significant differences at *P* < 0.05 and asterisks (**) denote significant differences at *P* < 0.01 byχ^2^ test (observed vs. expected). (JPG 7028 kb) Additional file 3: Figure S3. Olfactometer release rates of the full blend and the reduced blend. (JPG 1526 kb)
