# Supplementary figures and images for: Behavioural response of female Culex pipiens pallens to common host plant volatiles and synthetic blends
Source: Parasit Vectors. 2015 Nov 17;8:598. doi: 10.1186/s13071-015-1212-8 (PMC4650194; doi:10.1186/s13071-015-1212-8)

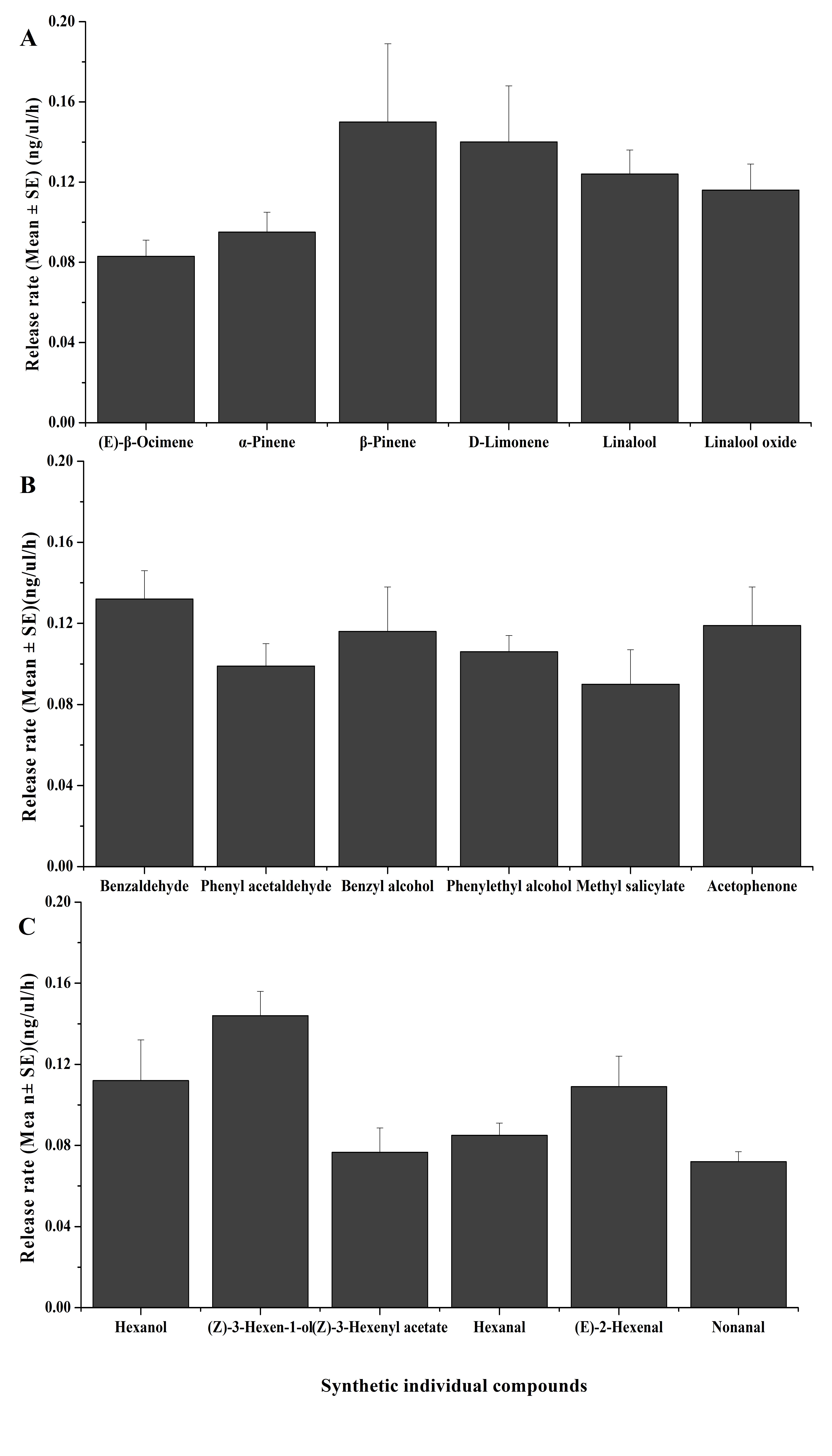

Supplement: Additional file 1: Figure S1. — Olfactometer release rates for optimal doses of 18 synthetic individual compounds. (JPG 2888 kb) [file 13071_2015_1212_MOESM1_ESM.jpg]

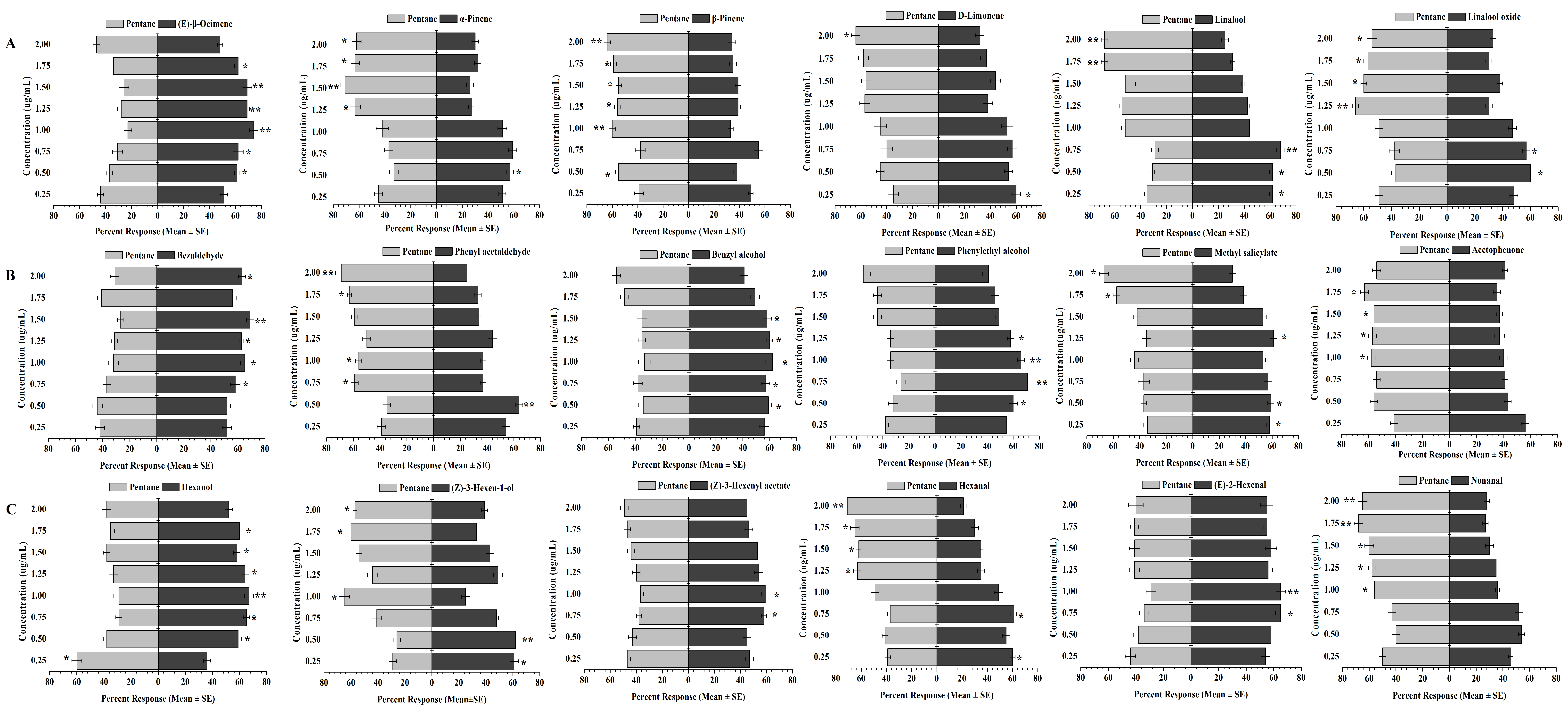

Supplement: Additional file 2: Figure S2. — Percent of mosquitoes responding to 18 individual compounds at different concentrations from monoterpenoids (A), benzenoids (B) and fatty acid derivatives (C). Asterisks (*) denote significant differences at P < 0.05 and asterisks (**) denote significant differences at P < 0.01 byχ2 test (observed vs. expected). (JPG 7028 kb) [file 13071_2015_1212_MOESM2_ESM.jpg]

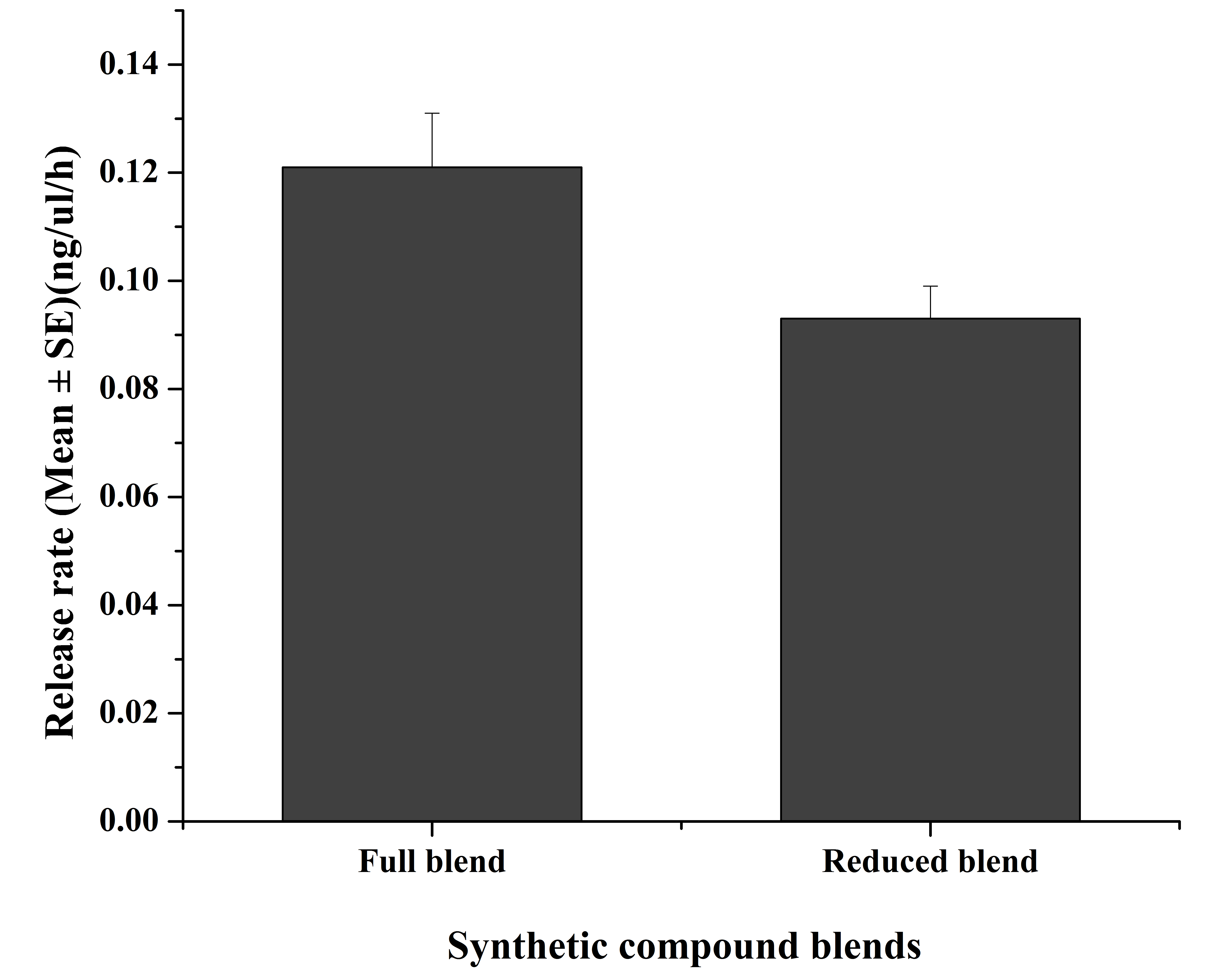

Supplement: Additional file 3: Figure S3. — Olfactometer release rates of the full blend and the reduced blend. (JPG 1526 kb) [file 13071_2015_1212_MOESM3_ESM.jpg]
